# Testing the Efficacy of Eptinezumab 100 mg in the Early Prevention of Chronic Migraine over Weeks 1 to 4: Prospective Real-World Data from the GRASP Study Group

**DOI:** 10.3390/jcm14248793

**Published:** 2025-12-12

**Authors:** Andreas A. Argyriou, Emmanouil V. Dermitzakis, Maria Chondrogianni, Aikaterini Foska, Dimitrios Rikos, Panagiotis Soldatos, Michail Vikelis

**Affiliations:** 1 Headache Outpatient Clinic, Department of Neurology, Agios Andreas State General Hospital of Patras, 26335 Patras, Greece; 2 General Clinic Thessaloniki, 54645 Thessaloniki, Greece; manolis.dermitzakis@gmail.com; 3 Second Department of Neurology, National and Kapodistrian University of Athens, School of Medicine, ‘Attikon’ University Hospital, 12462 Athens, Greece; mariachondrogianni@hotmail.gr (M.C.); dkfoska@gmail.com (A.F.); 4 404 Military Hospital, 41222 Larisa, Greece; rikosd@hotmail.com; 5 Independent Researcher, 24100 Kalamata, Greece; soldatosp@gmail.com; 6 Glyfada Headache Clinic, 16675 Athens, Greece; mvikelis@headaches.gr

**Keywords:** eptinezumab, early prevention, high-frequency chronic migraine

## Abstract

**Objectives**: This prospective, real-world study, designed by the Greek Research Alliance for Studying headache and Pain (GRASP), primarily aimed to examine whether a single administration of eptinezumab 100 mg can provide early therapeutic benefits over weeks 1–4 in preventing high-frequency chronic migraine (HFCM). **Methods**: We enrolled adults with HFCM (>23 monthly headache days—MHD) who had failed at least three preventive therapies and received a single infusion of 100 mg IV eptinezumab. Its efficacy was assessed using daily headache diaries over weeks 1–4 and at week 12, as well as with the Patients’ Global Impression of Change (PGIC) scale at week 4. Primary outcomes included ≥50% reduction in monthly headache days (MHD) through week 4 and early response at week 1. Secondary outcomes included changes in migraine severity, acute medication use, and incidence of most bothersome symptoms (MBS) accompanying headache. **Results**: A total of 39 HFCM patients were analyzed. Their mean age was 48.1 years, and most were female. More than half (*n* = 22; 56%) were fast-responders, showing >50% reduction in MHD at week 1. Early 50% response rates at week 1 favored CGRP-naïve patients, compared to prior non-responders. Likewise, significant improvement was observed early across all other efficacy domains through week 4, with sustained benefits through month 3. MBS, like photophobia and nausea, decreased notably, while osmophobia and allodynia improved less over weeks 1–4, post-eptinezumab. At week 4, 64.1% of patients reported meaningful overall improvement on PGIC. **Conclusions**: Eptinezumab rapidly prevented HFCM with clinically meaningful benefits seen early at week 1. Patients experienced sustained improvement in all measured clinical domains through week 4 and onward to month 3.

## 1. Introduction

Migraine is a debilitating neurological disease characterized by recurrent, moderate to severe throbbing headaches that can significantly impair quality of life. Symptoms commonly associated with migraine, coined as most bothersome symptoms (MBSs), include nausea or emesis, visual disturbances, and increased sensitivity to light, sound, and smell. Patients often experience prodromal signs, such as mood changes or fatigue, that precede the headache [1].

Chronic migraine (CM), which actually complicates episodic migraine and does not constitute a separate clinical entity, is clinically defined as usually severe headaches occurring 15 or more days per month (MHD) for 3 or more months, with at least eight days per month classified as migraine [2]. Compared to episodic migraine, it is generally perceived that CM is linked to substantially greater disability, elevated rates of comorbidity, and heightened direct and indirect expenditures [3].

This is partly because of the presence of cutaneous allodynia (another clinically significant MBS), recognized as a substantial indicator of central sensitization [4,5], markedly exacerbating pain sensitivity and the impairment linked to CM [6]. Indeed, recent studies highlight the role of central sensitization in the pathophysiology of CM, indicating a crucial link between neurobiological changes and the extent of various clinical manifestations [7]. Comorbid bruxism could also predispose to significantly more intense CM, in terms of headache severity [8]. Furthermore, the impact of CM extends beyond physical symptoms and profoundly affects daily life. People with CM often face significant psychological burden, including anxiety and depression, resulting from the unpredictability of their condition and the impact on their social and occupational functioning [9]. In addition, the socio-economic burden cannot be neglected as many individuals may experience decreased productivity, job loss, and increased healthcare costs [10]. However, there is evidence to suggest that high-frequency EM (HFEM) patients may exhibit a comparable level of disability and migraine-related burden to CM patients experiencing less than 23 MHD [11,12].

Monoclonal antibodies targeting calcitonin gene-related peptide (antiCGRP Mabs) have emerged as a significant advance in the treatment of migraine, providing distinct mechanisms, numerous benefits, and associated challenges. CGRP is a neuropeptide that plays a crucial role in the pathophysiology of migraine, functioning as a potent vasodilator and proinflammatory agent. The mechanism by which CGRP monoclonal antibodies operate involves inhibition of the binding of CGRP to its receptors, thereby attenuating migraine-associated pathways [13]. The main advantage of anti-CGRP Mabs is their effectiveness in reducing both the frequency and severity of migraine attacks, as evidenced by numerous clinical studies. These studies indicate that such treatment options can significantly improve the quality of life of patients, even those suffering from refractory CM [14]. Furthermore, the safety profile of anti-CGRP Mabs appears favorable, as they are generally well-tolerated with minimal adverse effects reported, therefore presenting a valid long-term management strategy [15,16].

Currently in Greece, there are four commercially available anti-CGRP Mabs, including fremamezumab, galcanezumab targeting the CGRP ligand, and erenumab targeting the CGRP receptor [17]. The fourth is eptinezumab, which is a humanized monoclonal antibody that has lately attracted attention for its role in the prevention of migraine, particularly due to its unique pharmacological properties [18]. It inhibits with high affinity and specificity CGRP, a neuropeptide that plays a critical role in the pathophysiology of migraine. By targeting the CGRP ligand, eptinezumab disrupts the signaling pathways generating migraine attacks [19]. Its intravenous formulation offers the advantage of 100% bioavailability and rapid onset of action because of immediate maximum concentration (Cmax within 30 min after its administration) [20]. As a result, some patients could experience relief within a few days after administration [21]. Various clinical trials demonstrated that eptinezumab is effective in reducing MHD, with a substantial number of both HFEM and CM patients exhibiting a significant reduction in migraine days and MBS frequency over weeks 1–12 [22,23,24,25,26]. This favorable benefit–risk ratio led to its approval for migraine prevention in patients with at least four monthly headache days, first by the US Food and Drug Administration in 2020 and later by the European Medicines Agency in 2022 [18].

However, considering the cyclical patterns of migraine with fluctuating response over time [27], it is difficult to comprehend how the rapid onset of eptinezumab action can reliably be assessed in HFEM or even in CM patients experiencing less than 23 MHD. As such, to control for this critical confounding variable, the primary aim of this real-world study, designed by the Greek Research Alliance for the Study of headache and Pain (GRASP), was to examine whether a single administration of eptinezumab 100 mg can provide early therapeutic benefits over weeks 1–4 in the prevention of patients with high-frequency chronic migraine (HFCM), exhibiting >23 MHD.

## 2. Materials and Methods

### 2.1. Study Design and Patient Selection

This open-label, prospective, multicenter, real-world study was conducted by headache specialists within the GRASP network. Eligible participants were consecutive adult patients diagnosed with high-frequency chronic migraine (HFCM), defined as >23 MHD, who had demonstrated inadequate response to more than three previous preventive treatments, including onabotulinumtoxinA, and were, as such, eligible to receive eptinezumab 100 mg, according to the national reimbursement requirements for high-cost therapies.

Migraine diagnosis followed the 2018 International Classification of Headache Disorders (ICHD-III) criteria [28]. Eligibility was verified using a protocol-specific screening checklist. All participants provided written informed consent, and the study protocol received Institutional Review Board approval in accordance with the Declaration of Helsinki. The study was part of the GRASP migraine registry and, therefore, was not separately registered as a public clinical trial.

Recruitment took place between September 2024 and September 2025 and included adults with confirmed HFCM for at least 6 months. Patients with or without aura or medication-overuse headache (MOH) were allowed to participate. MOH was defined as the use of one or more acute or symptomatic headache medications on ten or more days per month for triptans, ergots, combination analgesics or opioids, and on fifteen or more days per month for simple analgesics or nonsteroidal anti-inflammatory drugs, sustained for more than three months [28]. Eligibility required alignment with the approved therapeutic indication. Exclusion criteria comprised any contraindication to eptinezumab according to standard clinical practice and the approved Summary of Product Characteristics [29]. Patients with major psychiatric disorders, including disorders within the schizophrenia spectrum or other psychotic disorders. Participants with severe or uncontrolled obsessive–compulsive disorder were also excluded, as were those with active illicit substance use disorders.

### 2.2. Intervention

Patients received intravenous eptinezumab (Vyepti^®^, 100 mg/mL, Lundbeck A/S, Copenhagen, Denmark) administered at a fixed dose of 100 mg. Infusion was performed over 30 min in accordance with routine clinical practice. Eptinezumab 100 mg was administered as an intravenous infusion, diluted in 100 mL of 0.9% sodium chloride. Infusions were prepared using aseptic technique, inspected for particulate matter or discoloration, and administered through a standard intravenous line using a calibrated infusion pump over 30 min. The infusion set included a sterile, low protein-binding inline filter. Participants were monitored during the infusion and during a brief post-infusion observation period to assess for infusion-related reactions.

### 2.3. Efficacy Evaluation

Participants maintained a paper-based daily headache diary beginning at baseline (three months prior to first infusion) and continuing through follow-up assessments at weeks 1, 2, 3, 4, and month 3, post-treatment. Compliance was defined as documentation of at least 80% of total diary days. Patients were required to experience, overall, >23 MHDs. At least four of the MHDs had to occur within the last week prior to eptinezumab administration.

We longitudinally assessed the number of MHD; the number of MHD with peak pain intensity ≥ 5 on a 0–10 visual analogue scale (VAS 0–10) [30]; the monthly intake of acute migraine medications (MAI); and the frequency of MBS. MBS were collected in categorical yes/no format during baseline and each follow-up visit, including nausea/vomiting (sensation of impending emesis and involuntary gastric expulsion), photophobia (increased sensitivity to light), phonophobia (abnormal intolerance to sounds), osmophobia (excessive sensitivity or aversion to odors), and cutaneous allodynia defined as unpleasant or pain elicited by normally non-painful stimuli [31,32].

The primary efficacy endpoint was the proportion of responders achieving >50% reduction in MHD during weeks 1–4 and at week 12 compared with baseline. The proportion of patients achieving this threshold at week 1 (“fast responders”) was designated as a co-primary endpoint.

Secondary efficacy outcomes included longitudinal changes from baseline to week 12 in (i) total MHD; (ii) MHD with peak VAS ≥ 5; (iii) MAI frequency; and (iv) frequency of MBS. Patients’ perceived benefit and treatment satisfaction were assessed at week 4 using the Patient Global Impression of Change (PGIC) scale, a 7-point validated patient-reported outcome measure (1 = no change; 7 = considerable improvement) [33]. A PGIC score ≥ 5 was considered indicative of clinically meaningful improvement [34].

### 2.4. Statistical Analysis

Descriptive statistics were calculated according to the type of variable. Changes in primary and secondary efficacy outcomes from baseline through week 4 and week 12 were evaluated using analysis of covariance (ANCOVA), adjusted for baseline variables such as age, gender, comorbidity profile, and number of prior preventive treatment failures. All statistical tests were two-tailed, with a significance threshold of *p* < 0.05 unless specified otherwise. Statistical analyses were conducted using SPSS for Windows, version 27.0 (SPSS Inc., Chicago, IL, USA).

## 3. Results

All of our initially enrolled patients fully complied with filling in the headache diaries, and, as such, there were no missing data to handle. The mean patient age of the 39 participants who completed, mainly, the 4-week but also the 12-week follow-ups was 48.1 ± 12.5 years (range 18–68), and the majority were female (92.3%; *n* = 36). The median duration since migraine diagnosis was 27 years. According to ICHD-III criteria [28], 35 patients (89.7%) met the criteria for medication overuse headache (MOH) at baseline, and psychiatric comorbidities were also frequently observed. Prior to initiation of eptinezumab, 23 patients (*n* = 59%) were naïve to anti-CGRP monoclonal antibody treatment, whereas 16 patients (41%) had previously exhibited inadequate response to receptor- and/or ligand-targeting anti-CGRP monoclonal antibodies. Table 1 describes the demographic and clinical features of participants.

### 3.1. Changes Relating to Patients’ Primary and Co-Primary Clinical Outcomes over Time

A longitudinal analysis was conducted to evaluate the proportion of patients achieving more than 50% improvement across three key efficacy domains, including >50% response rate (RR), MHD with pain severity of VAS ≥ 5/10, and MAI. Before the initiation of treatment with eptinezumab, the baseline percentage values for all these three measures were high, i.e., 100% for the >50% RR, 80% (*n* = 31/39) for the MHD with pain severity of VAS ≥ 5, and 90% (*n* = 35) for MAIs. A noticeable early improvement at week 1 was evident, where all three measures decreased to a similar range of about 55–60% (*p* < 0.001). Specifically, >50% RR significantly dropped to 56% (*n* = 22), MHD with pain severity of VAS ≥ 5 to 60% (*n* = 23), and MAI to 55% (*n* = 21). Based on >50% RR outcomes at week 1, 22 of 39 patients (56%) were classified as fast responders, exhibiting substantial early therapeutic benefit following eptinezumab administration. Among these 22 fast-responders at week 1, 7/39 (18%) achieved ≥ 75% migraine response.

After week 1, a gradual upward trend was observed, reflecting ongoing improvement. At week 2, the corresponding percentages were 59% (*n* = 23), 66% (*n* = 26), and 62% (*n* = 24). By week 3, improvements continued, reaching 64% (*n* = 25), 70% (*n* = 28), and 69% (*n* = 27), respectively. At week 4, values remained consistent between 62 and 69%, indicating sustained benefit. Specifically, 62% of patients reached a reduction of more than 50% in headache frequency (*n* = 24). A similar improvement was documented for monthly headache days with pain intensity VAS ≥ 5, with 69% of patients meeting this threshold (*n* = 27). The same proportion, 69%, showed a greater than 50% decrease in their MAIs (*n* = 27). These favorable effects persisted through Month 3, with 67% for >50% RR (*n* = 27), 70% for MHD with VAS ≥ 5 (*n* = 28), and 70% for MAI (*n* = 28).

**Figure 1:**
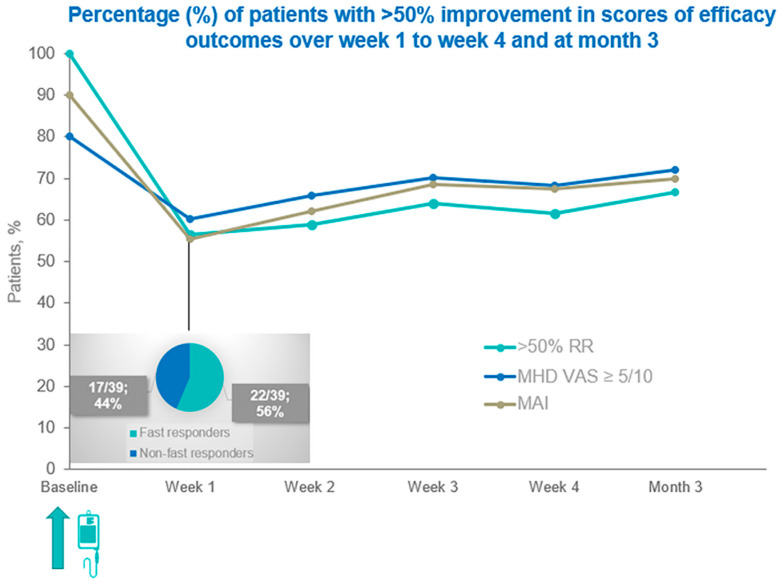
Percentage of patients with >50% improvement in efficacy outcome scores from week 1 through week 4 and at month 3. Figure 1 abbreviations: >50% RR: >50% response rates; MHD VAS ≥ 5: MHD with peak pain severity of VAS ≥ 5/10; and MAI: monthly acute medication intake.

Figure 1 illustrates changes in the percentage of patients who achieved greater than 50% improvement in efficacy outcome scores from week 1 through week 4 and at month 3. Embedded is the percentage of fast-responders at week 1, post-eptinezumab.

### 3.2. Changes in the Occurrence of Patients’ MBS over Time

Five distinct MBS were monitored to assess symptom-specific improvement: photophobia, phonophobia, nausea/emesis, osmophobia, and allodynia. At baseline, the highest reported rates were for photophobia (90%; *n* = 35), phonophobia (87%; *n* = 34), and nausea/emesis (77%; *n* = 30), while osmophobia (46%; *n* = 18) and allodynia (51%; *n* = 20) were less common.

By week 1, all MBS exhibited declines, indicating early symptomatic response to eptinezumab. Specifically, photophobia decreases slightly to 82% (*n* = 29/35), remaining the most frequently occurring MBS. Phonophobia dropped to 77% (*n* = 26/34), while nausea/emesis also declined to 67% (*n* = 20/30). The percentage of patients experiencing osmophobia and allodynia was reduced to 40% (*n* = 7/18) and 40% (*n* = 8/20), respectively.

At week 2, the downward trend continued for most MBS, though some showed signs of stabilization. Specifically, the rates of nausea/emesis were reduced to 64% (*n* = 19), photophobia to 77% (*n* = 27), while phonophobia (*n* = 26) remained steady at 77%, showing temporary stabilization. Osmophobia remained unchanged at 40% (*n* = 7), while allodynia (*n* = 6) declined further to 31%, becoming the lowest-improving symptom at this time point.

By week 3, further reductions were noted. Photophobia decreased to 59% (*n* = 21), phonophobia to 56% (*n* = 19), and nausea/emesis to 50% (*n* = 15). Osmophobia remained constant (40%; *n* = 7), and allodynia showed a minor rebound to 36% (*n* = 7). At week 4, rates plateaued, suggesting stabilization: photophobia increased slightly to 63% (*n* = 22), phonophobia declined to 51% (*n* = 17), nausea/emesis remained at 50% (*n* = 15), while osmophobia and allodynia were 33% (*n* = 6) and 31% (*n* = 6), respectively.

By month 3, the longer-term results showed that photophobia and nausea/emesis were stabilized at 63% (*n* = 22) and 41% (*n* = 12), respectively. Phonophobia continued to exhibit great improvements at 50% (*n* = 17). Osmophobia and allodynia decreased further to 28% (*n* = 5) and 25% (*n* = 5), reflecting lower sustained responsiveness.

**Figure 2:**
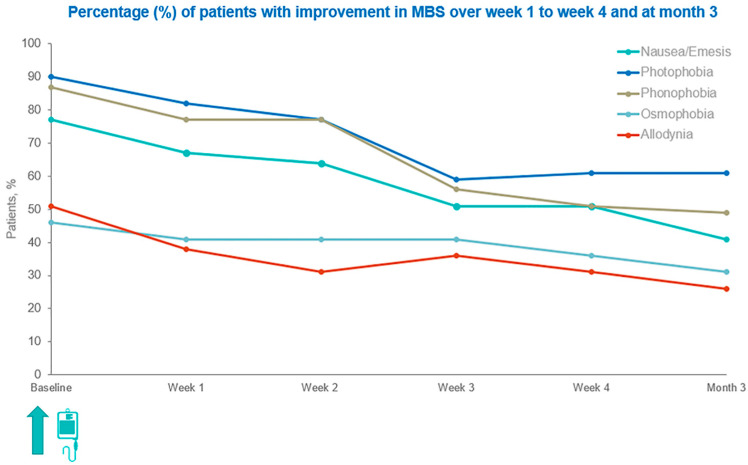
Percentage of patients with improvement in MBS from week 1 through week 4 and at month 3.

Figure 2 illustrates the percentage of patients who experienced improvement in MBS from week 1 through week 4 and at month 3.

Subgroup analysis stratified by prior exposure to anti-CGRP Mabs revealed that patients without previously being exposed to subcutaneous anti-CGRP Mabs (*n* = 23) were more likely to achieve at least a 50% reduction in MHD by week 1, compared with those (*n* = 16) who had previously failed other CGRP mAbs (16/23; 69.5% vs. 6/16; 37.5%, chi-square test: 0.04).

### 3.3. Patient Global Impression of Change (PGIC) at Week 4, Post-Eptinezumab

At week 4 following eptinezumab 100 mg preventive treatment, patients’ global impressions of their clinical condition indicated substantial perceived improvement. Overall, 48.7% (*n* = 19) of participants rated themselves as “much better” (score 6) or “very much better” (score 7) on the PGIC scale, while an additional 15.4% (*n* = 6) reported meaningful improvement (score 5). In total, 64.1% of the cohort experienced a clinically significant subjective improvement at this time point.

## 4. Discussion

Although a diagnostic category or threshold of more than 23 MHD is not currently formalized, we applied this cutoff to address the study objectives. Patients within this range represent a particularly severe subset of CM, characterized by near-daily headache and MBS occurrence, profound functional impairment, and the need for more intensive and individualized preventive treatment strategies. As such, the influence of this important confounding factor imposed by the cyclical pattern of migraine, mostly in HFEM, but also in low-frequency CM, was mitigated.

The findings of this real-world study demonstrated that a single administration of eptinezumab 100 mg can provide clinically relevant early and sustained therapeutic benefit in patients with HFCM through weeks 1–4. In the subgroup analysis, patients who had not previously received subcutaneous anti-CGRP Mabs showed a notably stronger early response to treatment. By week 1, nearly 70% of these patients achieved at least a 50% reduction in MHD, compared with only 37.5% among those who had previously failed subcutaneous anti-CGRP mAbs. This difference reached statistical significance (chi-square *p* = 0.04), suggesting that prior exposure or treatment failure with anti-CGRP mAbs may reduce the likelihood of an early therapeutic response. The rapid onset of action observed as early as week 1 supports previous clinical trial evidence indicating that eptinezumab might exert a beneficial effect soon after exposure. No gender effects on our findings can be substantiated, because the vast majority of participants (92.3%) included in the study were female.

The PROMISE-1 trial assessed intravenous eptinezumab at different doses for the prevention of episodic migraine. Eptinezumab produced a statistically significant and clinically meaningful reduction in migraine frequency during weeks 1 to 12 after the first infusion at a dosage of 100 mg in 212 patients. Preventive benefits were detectable as early as day 1 [22]. A prespecified, alpha-controlled analysis in the phase 3 PROMISE-1 and PROMISE-2 trials showed that both 100 mg and 300 mg eptinezumab achieved significant preventive efficacy by day 1 after infusion. Reductions in monthly migraine days exceeded placebo in both episodic and chronic migraine cohorts. These findings indicate that eptinezumab provides a rapid and sustained preventive effect measurable from the first day of treatment [23]. In the PROMISE-2 trial, both 100 mg and 300 mg eptinezumab resulted in a significant reduction in monthly migraine days starting the day after infusion and continuing through week 12. Patients receiving eptinezumab were also more likely to achieve a ≥75 percent response during weeks 1 to 4 compared with placebo, with odds ratios of 2.4 (95% CI 1.7–3.5) for 100 mg and 3.2 (95% CI 2.2–4.6) for 300 mg [24]. Finally, another randomized, double-blind, placebo-controlled phase 3 trial investigated whether intravenous eptinezumab 100 mg, given within six hours of migraine onset, improves acute outcomes in adults with episodic migraine. Eptinezumab led to faster achievement of headache pain freedom and resolution of the most bothersome symptom compared with placebo, with significant effects evident as early as two hours after treatment. Fewer patients required rescue medication within 24 h, suggesting a meaningful clinical benefit [25].

Compared with the published trial findings, our real-world data in HFCM appear broadly consistent with the rapid onset and sustained preventive effect reported for eptinezumab in controlled settings, yet with some notable differences attributable to disease severity and study design. In PROMISE-1 and PROMISE-2, preventive efficacy emerged from day 1 and remained stable through week 12, with clear separation from placebo across episodic and chronic migraine cohorts [22,24]. Similarly, in our cohort, preventive benefit was also evident early and persisted throughout follow-up, despite the significantly higher baseline headache burden (>23 MHD) and greater clinical refractoriness. Response rates in our sample, therefore, support the generalizability of the early-onset effect even in a more resistant migraine phenotype. In contrast to the acute-administration trial demonstrating accelerated pain resolution during an evolving attack [25], our study did not assess acute response or time-to-pain freedom. However, the rapid preventive onset observed in both contexts reinforces the pharmacodynamic advantage of intravenous delivery and the potential clinical value of early reduction in migraine activity.

Nonetheless, the present cohort differs substantially from typical CM samples because participants reported more than 23 MHD at baseline, and most had long disease duration, medication overuse, and prior preventive treatment failures. These clinical characteristics make this population particularly difficult to treat and increase the relevance of the observed response trajectory.

The high proportion of week-1 responders, with 56% achieving more than 50% reduction in headache frequency, supports the hypothesis that early response can be reliably detected in this more severe and clinically stable population. Earlier work raised questions about whether fluctuating migraine patterns may obscure early efficacy signals in episodic migraine or lower-frequency CM [22,23]. In contrast, the high and stable baseline headache burden in this cohort reduces the influence of natural variability and strengthens the interpretation of early efficacy. This aligns with the mechanistic rationale that intravenous administration and full bioavailability facilitate rapid therapeutic effect [35].

The progressive improvement from week 1 to week 4 and the persistence of treatment benefit at month 3 indicate sustained efficacy. The pattern of response across primary outcomes, including MHD, MAI, and pain severity, was consistent, suggesting that the observed improvements reflect a broad therapeutic effect rather than partial symptomatic relief. A similar trend was evident in the reduction in MBSs. Photophobia, phonophobia, and nausea remained the most prevalent symptoms throughout treatment, but their progressive decline suggests an influence beyond attack frequency modulation, possibly related to central anti-nociceptive mechanisms [36].

MOH was highly prevalent at baseline in our study. Although we did not stratify treatment response based on medication overuse status, the observed improvements suggest that eptinezumab may provide clinical benefit even without prior structured withdrawal. This observation requires cautious interpretation and warrants further controlled evaluation because medication overuse has been historically associated with poorer response to preventive therapy [37]. The PGIC data reflect a strong concordance between objective improvements and patient-reported benefit. By week 4, almost half of the participants described themselves as “much better” or even “very much better,” and another group reported that their condition had clearly improved. Overall, nearly two-thirds of participants perceived meaningful improvement after four weeks, complementing the objective improvements seen in headache frequency, but also likely underscoring the clinical relevance of the magnitude and timing of response.

Our study has several strengths, including its prospective design, strict eligibility criteria aligned with national reimbursement policy, high diary compliance, study of difficult-to-treat CM, and a consistent follow-up schedule. Hence, our real-world setting enhances clinical generalizability. Limitations include the absence of a placebo or standard-care control group, while the relatively small sample size prevents exclusion of confounding factors (e.g., natural migraine cyclic remission, regression to the mean). The open-label design may also introduce expectation bias, although this is unlikely to fully explain the early and sustained magnitude of clinical benefits observed. Moreover, our results apply to treatment-resistant HFCM without statistical adjustment, and, as such, validation is needed in broader populations. Finally, although the binary structure in assessing MBS aligns with the trial’s predefined endpoints, we might acknowledge that this approach might limit the ability to quantify the magnitude of corresponding improvement. As such, further studies, incorporating continuous severity MBS metrics, are warranted.

## 5. Conclusions

To conclude, our results demonstrate that a single infusion of eptinezumab 100 mg is effective in a difficult-to-treat subgroup of CM patients with the highest headache frequency. The rapid onset of eptinezumab action may support its use in patients requiring a timely clinical benefit, especially those with persistent disability and multiple prior treatment failures. Patients naïve to subcutaneous anti-CGRP Mabs showed a markedly stronger early response, strongly supporting the view that previous anti-CGRP Mab failure may diminish early treatment effectiveness. Nonetheless, comparative effectiveness studies are needed to better define the predictors of early response, the role of fast responders, and the clinical implications of early symptom improvement in the prevention of CM with eptinezumab.

## Figures and Tables

**Table 1:** Demographic and clinical characteristics of participants at baseline.

Participants *n* = 39	
Variable	*n* (%)
**Gender**	
Females	36 (92.3)
Males	3 (7.7)
**Age ± SD (range)**	48.1 ± 12.5 (18–68)
**Previous lines of prophylactic medications**	
median value (range)	4 (3–10)
**Years with chronic migraine diagnosis**	
median value (range)	27 (9–40)
**Psychiatric comorbidities**	
No	9 (23.1)
Anxiety disorder	10 (25.6)
Depression	6 (15.4)
Mixed anxiety and depression disorder	14 (35.9)
**Medication overuse headache**	
Yes	35 (89.7)
No	4 (10.3)
**Aura**	
Yes	12 (30.8)
No	27 (69.2)
**Prior anti-CGRPs Mabs exposure**	
Naive	23 (59.0)
Anti-CGRP-receptor Mab and/or Anti-CGRP-ligand Mab	16 (41.0)

## Data Availability

The data that support the findings of this study are available from the corresponding author upon reasonable request.
